# Compliance of Pharmaceutical Manufacturing Companies to Good Manufacturing Practices in Heating, Ventilation, and Air-Conditioning Systems: The Case of Local Ethiopian Firms

**DOI:** 10.1155/adpp/6109415

**Published:** 2024-12-07

**Authors:** Desta Tune Tibesso, Tesfaye Gabriel, Tamrat Balcha Balla, Anteneh Belete

**Affiliations:** ^1^Department of Pharmaceutics and Social Pharmacy, School of Pharmacy, College of Health Sciences, Addis Ababa University, Addis Ababa, Ethiopia; ^2^Department of Pharmaceutical Technology, School of Pharmacy, College of Health Sciences and Medicine, Wolaita Sodo University, P.O. Box 158, Wolaita Sodo, Ethiopia; ^3^Center for Innovative Drug Development and Therapeutic Trials for Africa (CDT-Africa), College of Health Sciences, Addis Ababa University, Addis Ababa, Ethiopia

**Keywords:** Ethiopia, good manufacturing practice, HVAC system, implementation status, local pharmaceutical manufacturing companies

## Abstract

**Background:** Good manufacturing practice (GMP) is a part of quality management that maintains product quality and manages it according to the criteria of fitness for use. The heating, ventilation, and air-conditioning (HVAC) system comprises the equipment, technology, and procedure that ensure product quality through maintaining heat, ventilation, and coolness of pharmaceutical manufacturing firms. The aim of the study was to evaluate the GMP compliance of HVAC systems and assess the opportunities and challenges of improving these systems in pharmaceutical manufacturing companies in Ethiopia.

**Methods:** The study was conducted in eight local pharmaceutical manufacturing companies in Ethiopia from April 20 to August 30, 2021, by using a concurrent mixed-method approach to evaluate the implementation of GMP in HVAC systems. The pharmaceutical firms were directly observed by using a structured and standard observational checklist that was adopted from the WHO minimum GMP requirements. In order to understand the challenges and opportunities, face-to-face interviews with key informants using a purposive sampling technique were conducted.

**Results:** The study findings revealed that the local pharmaceutical companies applied for 67.1% of the GMP requirements of the HVAC systems. The GMP implementation status in the basic quality features of HVAC systems of local pharmaceutical companies was 75% on premises, 70.67% in HVAC system design and maintenance practices, 56.25% in product protection, 61.25% in environmental protection, and 75% in cross-contamination prevention. The primary obstacles to implementing GMP in the HVAC systems were a shortage of skilled professionals, HVAC system spare parts, and foreign currency besides poor practice of HVAC system calibration.

**Conclusion:** The study revealed that the local pharmaceutical manufacturing companies did not adhere to GMP standards in HVAC systems.

## 1. Introduction

Good manufacturing practice (GMP) is a part of quality management that maintains product quality and manages it according to the criteria of fitness for use [1]. It requires all manufacturing processes to be clearly defined, reviewed on a regular basis, and show systematic production of pharmaceutical products in order to ensure quality [2, 3]. The essence of GMP emanates from the tenet that the end-point quality testing is insufficient to ensure the quality of pharmaceutical products, and it must be ensured at each of the manufacturing steps [4]. Compliance with GMP is an integral attribute of the pharmaceutical quality system. It is ensured through the trained and responsible personnel, equipment and facilities that are fit for their intended purpose, adequate documentation system, quality manufacturing processes that ensure consistent production, and investigation and correction of quality defects and deviations [5, 6].

The heating, ventilation, and air-conditioning (HVAC) system includes the equipment, technology, and process that maintain a pharmaceutical manufacturing company's heat, ventilation, and coolness. It plays an important role in guaranteeing product quality [7]. To guarantee indoor air quality in pharmaceutical manufacturing facilities, the HVAC system maintains a controlled unidirectional airflow, defined air velocity, specific air movement patterns, and tightly regulated temperature and relative humidity levels [8–10]. Filtration and HVAC systems play key roles in the quality of supplied air by preventing exhaust gases, vapors, and airborne particles, including microbes, from entering the manufacturing plant [11, 12]. Contamination and cross-contamination are the outcomes of poor HVAC system design and maintenance [13, 14].

The manufacture of pharmaceuticals should rigorously adhere to the GMP guideline set by WHO. Hence, this is an in-depth study to evaluate the GMP implementation status of HVAC systems in Ethiopian local pharmaceutical manufacturing companies. The study primarily focused on evaluating the quality aspects of HVAC systems such as the premises, design, and maintenance practices. Besides, the requirements for protecting products, preventing cross-contamination, and protecting environments as well as assessment of challenges and opportunities to adhere to GMP in HVAC systems were addressed.

## 2. Methodology

### 2.1. Study Area and Period

There are 11 pharmaceutical manufacturing companies having 2160 employees and a 20% turnover rate. Of these companies: seven, three, and one are located in Addis Ababa, Oromia, and Amhara regions, respectively. These companies manufacture finished products such as antibiotics, gastrointestinal medications, antihistamines, anthelminthics, cardiovascular medications, antidiabetic agents, antiprotozoans, analgesics, respiratory medications, vitamins, minerals, large-volume parenteral products, dermatological preparations, and veterinary vaccines. One of these companies produces empty hard gelatin capsule shells. The present study was conducted in eight local pharmaceutical manufacturing companies from April 20 to August 30, 2021.

### 2.2. Study Design and Population

A concurrent mixed-method study design was used. All the local pharmaceutical manufacturing companies that were licensed to manufacture pharmaceutical products by the Ethiopian Food and Drug Administration (EFDA) and personnel working in the companies comprised the study population. The companies operate both in domestic and international markets.

### 2.3. Eligibility Criteria

Local pharmaceutical manufacturing companies licensed by EFDA were included in the study and key personnel were included in the key informant interviews. The companies which were closed during the data collection period and small-scale local pharmaceutical manufacturing companies were excluded.

### 2.4. Sampling Techniques

Participants for key informant interviews were selected by using a purposive sampling technique. The selection was based on their responsibilities in the companies, namely, general manager, production manager, quality assurance manager, or technical assistant manager. In addition, the pharmaceutical firms were observed to assess their GMP implementation status with respect to HVAC systems.

### 2.5. Data Collection Tools and Procedures

The quantitative data collected by professionals who had experiences of 3-4 years in HVAC systems by using a structured and standard observation checklist that was adopted from WHO minimum GMP requirements in HVAC systems were used. The data collection was performed using actual observation of practices complemented by consulting technical experts of the companies. The consultation was based on the items on the standard observation checklist and the processes in each pharmaceutical company were evaluated for 3 days.

For the qualitative part of the study, key informant face-to-face interviews were held using a semistructured questionnaire. The respondents included key personnel such as quality assurance manager, production manager, technical assistant manager, or general manager of a company. The interview was conducted in English and voice recorded. The interviews were continued until data saturation was reached.

### 2.6. Study Variables

Implementation of GMP in the HVAC system was the dependent variable. GMP requirements in HVAC systems including premises, design and maintenance practices, product protection, environmental protection, and prevention of cross-contamination were the independent variables.

### 2.7. Data Quality Management

The standard checklist and semistructured interview questionnaires were evaluated by experts to improve data collection tools and ensure data quality. Then, some questions were modified and rephrased. After adopting the final version, the questionnaires were prepared in hard copy. To ensure that the data were comprehensive, organized, consistent, and correct throughout the data collection, each questionnaire was reviewed for clarity and completeness on the day of the data collection. The data entry and validation were performed using EpiData Version 3.1 software. Prior to data analysis, data cleaning was performed to check missing values and discrepancies for quantitative data.

Each interview transcript was read and reread for qualitative data in order to have an overall understanding and familiarity with the details. Then, the first theme framework's unit was identified and developed. The initial themes were used as guiding tools to discover more inclusive thematic concepts, ensuring a richer and more detailed understanding of the data. Finally, interview transcription was coded using the already developed thematic framework.

### 2.8. Data Analysis

Descriptive statistical analysis was used for quantitative data using SPSS Version 25.0 and the findings were organized and tabulated using frequency tables along with percent distributions. On the other hand, the collected field notes and audio-recorded transcripts were organized to address the research purpose. Each audio interview transcript was imported into the computer and using the play–pause button, the audio was typed on the computer in the English language. Then, coding and thematic analysis of the qualitative data were performed using Open Code Version 40.3 software.

### 2.9. Ethical Considerations

Ethical clearance was obtained from the Ethical Review Board of the School of Pharmacy, College of Health Sciences, Addis Ababa University on March 15/2021 with Ref. no: ERB/SOP/310/13/2021. Permission was also obtained from all the local pharmaceutical manufacturing companies. The management staff of the pharmaceutical companies first consented. After getting permission, key personnel and technical experts were apprised of the study's purpose. The purpose of the study was disclosed to the study participants and they were asked to participate voluntarily. Companies and participants were informed that the data they provided would be considered confidential and neither study participants nor their firms were disclosed during the analysis.

## 3. Results

### 3.1. Quantitative Findings

#### 3.1.1. Premises

The implementation of GMP in HVAC systems with respect to premises among the local pharmaceutical companies is presented in Table 1. All of the assessed companies had HVAC systems that were parts of the original design of the facilities and seven of the companies had airlocks with a differential pressure cascade. Although the basic requirements for GMP implementation on the premises include diagrams depicting the pressure cascade, airflow direction, and flow route of personnel and materials, only five of the companies fulfilled these standards.

#### 3.1.2. Design and Maintenance Practice of HVAC Systems

The study findings show that four of the companies showed a lack of the required capacity to guarantee the HVAC system's performance, and six of them had a lack of risk management requirements. Similarly, four of the companies did not have documentation support for the capacities that had been determined in the installation records' system. Still, four of the companies failed to provide staff training following installation, and five of the companies had a lack of test protocol. Seven of the companies reported that they had the validation master plans (VMPs) that specify the requirements for the HVAC systems. Only two of the companies had efficient air distribution and flow patterns, whereas four companies had set room recovery rates for their HVAC systems. Seven of the companies provided HVAC system qualification manuals and scheduled plans of preventive maintenance. They had also changed their HEPA filters and evaluated filters for leaks. Six of the companies had also determined the timeframe between tests and requalification of HVAC systems as can be seen in Table 2.

#### 3.1.3. Protection of Materials and Products

The study revealed that the air filtering systems, air change rates, and strategies for minimizing internal contaminants in three of the companies were ineffective. Only three of the companies appeared to protect the products and materials from contamination and cross-contamination across the entire manufacturing process. With regard to air filtration, four of the local pharmaceutical companies effectively filtered the air supply to remove external contaminants and controlled airborne contaminants as shown in Table 3.

#### 3.1.4. Protection of Environment

The GMP in HVAC systems' implementation concerning environmental protection was evaluated. Accordingly, only three of the companies had to efficiently filter the exhaust air before releasing air into the environment. Only two of the companies utilized standard EN779 filters for less toxic powders and standard EN1822 HEPA filters for toxic compounds to treat exhaust air (Table 4).

#### 3.1.5. Prevention of Cross-Contamination

This study revealed that GMP requirements in HVAC systems were to prevent cross-contamination in domestic pharmaceutical manufacturing firms. In terms of the products handled and the level of protection, six local pharmaceutical manufacturing companies performed pressure cascade assessments. The pressure cascade regime and airflow direction required for processing the product were available in seven of the companies. Five of the seven companies used a pressure cascade negative to ambient pressure to manufacture potent products as mentioned in Table 5.

#### 3.1.6. Overall Implementation of GMP in HVAC Systems

As shown in Table 6, 75% of the premises and prevention of cross-contamination GMP requirements in the HVAC system were implemented, while 70.67% of the GMP requirements were implemented in the design and maintenance practice of HVAC systems in local pharmaceutical manufacturing companies. The implementation level of GMP standards in the HVAC system for product protection requirements in local firms was 56.25%. Then again, the GMP HVAC systems' implementation concerning environmental protection requirements was 61.25%.

On the other hand, the study findings show that the GMP in HVAC system implementation status in local pharmaceutical firms was 67.1%. One of the local pharmaceutical companies had a very low (5.8%) overall GMP implementation status in the HVAC system, while two local pharmaceutical companies had 98.1% GMP implementation status in the HVAC system as noted in Table 6.

### 3.2. Qualitative Findings

#### 3.2.1. Sociodemographic Characteristics of Respondents

The sociodemographic characteristics of respondents in the local pharmaceutical manufacturing companies are shown in Table 7. Seven study participants were men, five of whom were between the ages of 36 and 45, six of whom were quality assurance managers, and three of whom had been engaged in their current job for more than 10 years. Six of the respondents had a pharmacy educational background with five of them having MSc degrees.

#### 3.2.2. The Thematic Area Emerged From the Study

In this study, there were four major themes emerged from the data. This theme includes the perceived implementation status of GMP in HVAC systems, challenges to implementing GMP in the HVAC systems, relevant stakeholders to mitigate challenges, and opportunities to implement GMP in the HVAC systems.

##### 3.2.2.1. Theme One: Perceived Implementation Status of GMP in HVAC Systems

The present study disclosed the perceived implementation status of GMP in the HVAC system. Concerning GMP in this system, four respondents stated that our company has a moderate level of GMP implementation in the HVAC system. However, the two respondents said that the HVAC system's GMP implementation and maintenance are subpar in our companies. On the other hand, two respondents said that the implementation status of GMP in the HVAC systems in our companies complies with the standard GMP requirements to ensure contamination prevention and protection of the environment, products, and personnel.

##### 3.2.2.2. Theme Two: Challenges to Implement GMP in HVAC Systems

The current study identified the major challenge to implementing GMP in the HVAC systems for local pharmaceutical manufacturing companies. Seven of the key informants state that a shortage of skilled human resources, HVAC system spare parts, HVAC system calibration practices, and a dearth of foreign currency are the major hindrances to implementing GMP in the HVAC system. They added that the greatest difficulties are scarcity of skilled human resources and spare parts.

However, a 36 year-old male key respondent stated that political and economic upheavals were the main challenges to implementing GMP in the HVAC systems for local pharmaceutical manufacturing companies.

##### 3.2.2.3. Theme Three: Relevant Stakeholders to Mitigate Challenges

This theme was developed from key respondent data about relevant stakeholders to mitigate the challenges in local pharmaceutical manufacturing companies. All of the key informants indicated that in order to reduce the challenges of GMP in the HVAC system, the National Metrology Institute should increase its capacity, the government should promote the import of spare parts, and the universities should cooperate with pharmaceutical companies. Furthermore, a 36 year-old male MSc Pharmacy professional who is a key respondent of the company states that the relevant stakeholders mitigate the challenges in the following manner:

To address the challenges, especially the economic consequences relevant to political instability, prioritizing political stability is essential.

##### 3.2.2.4. Theme Four: Opportunities to Implement GMP in HVAC Systems

This theme is inductively developed from the key respondent's responses about the opportunities of local pharmaceutical manufacturing companies. Seven of the respondents stated that the opportunities for local pharmaceutical manufacturing companies were favorable government support for the industry, preference for local companies, and assistance and training from the EFDA and the other stakeholders.

## 4. Discussion

To prevent cross-contamination and to provide environmental, personnel, and product safety in pharmaceutical firms, the architecture of the premises and the HVAC system should be rigorously considered [15, 16]. According to WHO, premises' architectural design and HVAC systems have a close relationship. The location, design, construction, adaptation, and maintenance of the premises must be performed in accordance with the corresponding operations to be carried out [17]. The layout and design of the premises must be in such a way as to avoid cross-contamination, dust accumulation, and any other negative effects on the quality of the products [18]. However, in this study, 75% of the local pharmaceutical companies implemented GMP in HVAC systems' requirements on premises. One of the weaknesses was that most local pharmaceutical manufacturing companies were old and in need of renovations. They had poor maintenance practices of the premises.

The major quality comprehensive schematic diagram that specifies pressure cascade, air flow direction, and flow route for persons and materials improves the quality of pharmaceutical products [7]. Despite this, three local pharmaceutical companies did not meet these standards. This was owing to the lack of rigorous inspections of these facilities and the old pharmaceutical companies with old premises had paid little attention to the GMP design of their process, personnel, effluent airflow patterns, or efficient space arrangements.

GMP in HVAC system services encompasses a wide range of concerns starting with the selection of construction materials and finishes; the flow of equipment, personnel, and products; the determination of critical parameters such as temperature, humidity, pressures, filtration, and airflow parameters, and the classification of clean rooms [9]. Throughout the life cycle, design and maintenance practices in HVAC systems should be managed. The system should be designed to regulate temperature and relative humidity, indicate the company's operational status, and limit the amount of viable and nonviable particle exposure to pharmaceutical companies [14]. The implementation status of design and maintenance practices in this study was 70.67%. The principal issues with the HVAC systems of the local companies were their reliance on the design and maintenance practices of foreign expertise. As a result, many local companies have not upgraded their HVAC design and maintenance systems. These circumstances significantly increased the expense of HVAC systems' maintenance and hindered the planned transfer of technology. The demand for cost and sophistication of technology led to an increase in the complexity of HVAC system requirements for the local pharmaceutical manufacturing companies.

According to Anyakora et al. [19], pharmaceutical firms implement the risk management principles that are specified for the design and the minimal capacity that ensures the HVAC system. Pharmaceutical companies assess risks to determine the level of validation necessary and the cleanliness of the rooms [20]. Quality risk management is one of the most important responsibilities in the framework of the pharmaceutical manufacturing industry [21]. Six of the local companies in this study were unable to implement risk management principles. The reasons were fear, lack of resources, an excessive number of risks identified, and a lack of regulatory guidance for local pharmaceutical manufacturers.

Starting materials, intermediate products, in-process materials, finished products, primary packaging, and equipment exposure to the environment should determine the cleanliness of areas to ensure the quality of pharmaceutical products [16]. However, 56.25% of the GMP in HVAC system requirements were implemented for product protection in local pharmaceutical companies. Low levels of product protection might be the impact of validation flaws, lack of interpretation and consistent evaluation, and monitoring mechanisms as a result of lax regulatory control, ineffective HVAC systems, and subpar design of premises.

According to Chaudhari and Sarje, environments should be protected from pollutants such as dust, airborne microbes, aerosol particles, and chemical vapors [22]. The current study finding showed that protection of the environment in local companies was only 61.25%. The environmental protection of the companies was still poor, most likely because of personnel negligence, lack of expertise, and awareness or the low priority given to environmental protection by the companies.

According to Eyob, in two local pharmaceutical manufacturing companies, the compliance with GMP standards for preventing cross-contamination was 90.3% and 77.2% [23]. Local pharmaceutical companies in the present study had a cross-contamination prevention rate of 75%. The lack of effective cross-contamination control might be caused by a variety of circumstances. These include the manufacturing of pharmaceutical items in shared facilities, less regulatory scrutiny, and organizational and technological flaws in the HVAC system.

The WHO states that standards of pharmaceutical manufacturers should not fail to meet GMP requirements. GMP compliance is required at a minimal level to guarantee that the pharmaceutical products meet the quality, safety, and efficacy requirements [16].

Incentives offered by the Ethiopian government for domestic pharmaceutical manufacturers include tax-free loans of up to 70% for new companies and 60% for upgrading. In addition, spare parts with up to 15% custom duty exemption as well as 100% custom duty exemption on imported goods such as manufacturing plant machinery, equipment, and construction materials are among the incentive packages [24]. According to Beedemariam et al., the government gives precedence to the local manufacturers besides the tax-free import of raw materials and equipment and the training support programs by EFDA [25]. This study revealed that most of the respondents identified the aforementioned opportunities. Even though the government provided the mentioned opportunities, the local pharmaceutical manufacturers were still suffering from a lack of foreign currency, skilled human resources, coordination, and communication with regulatory authorities and other stakeholders regarding the strategic directions in supporting the local companies. There was also a lack of competence and awareness of pharmaceutical shareholders to encourage local firms.

According to the present study, 67.1% of the local pharmaceutical manufacturing companies were in compliance with the basic quality features of GMP requirements in HVAC systems. One of the companies had a very low level of GMP implementation in HVAC systems. Similarly, most of the respondents stated that local pharmaceutical manufacturing companies had a moderate level of implementation of GMP in HVAC systems. These indicate that the local pharmaceutical manufacturing firms did not adhere to the GMP standards. This might be due to too many GMP requirements of the HVAC systems that overwhelmed the firms, forcing them to operate outside of GMP standards. This might in turn be because of a lack of financial, technical, and skilled human resources, as well as a high turnover of technical staff. In addition, there were concerns with the management systems of these firms, the dearth of GMP training facilities, the lack of qualified equipment calibration and maintenance facilities, and the weakness of their links to academic institutions [26].

The present study showed that local pharmaceutical companies had low GMP implementation status in HVAC systems and faced many obstacles. Most of the respondents stated that lack of skilled manpower, lack of spare parts, lack of calibration practices, and dearth of foreign currency were the major impediments. Beedemariam et al. and Birhanu and Ketele showed comparable challenges with the present study [25, 27]. According to Beedemariam et al., the main threats to the local companies were the shortage of foreign currency and the lag in importing raw materials and equipment. Similarly, in the literature, high production costs, lack of innovation and technology transfer, limited investment in research and development, shortage of skilled labor, inadequate infrastructure and manufacturing equipment, as well as limitations of hard currency and overall coordination between pharmaceutical manufacturers and other stakeholders, are the main challenges [25].

This shows that the efforts by the government and regulatory bodies to address these issues have not significantly improved the system because the transfer of knowledge and technology takes time. These are the most serious threats to local pharmaceutical companies to comply with cGMP.

### 4.1. Limitations of the Study

The study was not proposed to provide a comprehensive evaluation of cGMP implementation in local pharmaceutical manufacturing companies and it is forwarded only to local pharmaceutical manufacturing companies, which were included in the study. Moreover, due to the lack of previous reports on the status of GMP implementation in HVAC systems in local Ethiopian pharmaceutical manufacturing companies, this study could not assess the progression or regression of the performance of these firms in terms of implementing GMP in HVAC systems.

## 5. Conclusions

The study revealed that the local pharmaceutical manufacturing companies failed to adhere to cGMP minimum requirement standards in HVAC systems. Lack of skilled manpower, lack of HVAC system spare parts, weak practice of HVAC systems' calibration, and dearth of foreign currency were the major impediments to implementing and maintaining GMP in HVAC systems. There was favorable government support for the pharmaceutical industry, namely, the provision of incentive packages to the local manufacturers and technical support from EFDA and the other stakeholders. The findings of this study are relevant to the regulatory authority to work on strengthening pharmaceutical manufacturing regulations and creating institutes for innovative pharmaceutical manufacturing for spare part imports and for the calibration of HVAC systems.

### 5.1. Recommendations

Pharmaceutical manufacturing companies need to be diligent in qualifying, requalifying, and upgrading existing HVAC systems. In addition, the regulatory authority should endorse strengthening pharmaceutical manufacturing regulations, creating a public–private institute for innovative pharmaceutical manufacturing, for spare part imports and for calibration of the HVAC system. The government should provide the pharmaceutical industry financial incentives to modernize its technology and encourage global collaboration to hasten the adoption of emerging technologies.

## Figures and Tables

**Table 1:** Implementation of GMP in HVAC systems on the premises in local pharmaceutical manufacturing companies, 2021.

GMP in HVAC systems on premises requirements	Yes *N* (%)	No *N* (%)
Adequate airlocks in the company	7 (87.5)	1 (12.5)
Design of the HVAC system closely coordinated with the design of the company	8 (100)	0 (0)
Detailed diagrams depicting pressure cascades, air flow directions, and flow routes for personnel and materials	5 (62.5)	3 (37.5)
Appropriate personnel and materials movement	4 (50)	4 (50)

**Table 2:** Implementation of GMP on the design and maintenance practice of HVAC systems among local pharmaceutical manufacturing companies, 2021.

Design and maintenance practices of HVAC system requirements	Yes *N* (%)	No *N* (%)
HVAC system has sufficient capacity	4 (50)	4 (50)
Risk management principles applied	2 (25)	6 (75)
Intake and exhaust air terminal positioned in the manner of preventing cross-contamination	8 (100)	0 (0)
Air conditions and limits for temperature, relative humidity, and air cleanliness specified	7 (87.5)	1 (12.5)
Room recovery rate (clean-up) of the HVAC system specified	4 (50)	4 (50)
Air distribution and airflow patterns appropriate and effective	2 (25)	6 (75)
Appropriate alarm system	7 (87.5)	1 (12.5)
Failures of HVAC components assessed	6 (75)	2 (25)
Installation record system documented	2 (25)	6 (75)
Training provided to personnel for installation and operation	4 (50)	4 (50)
Qualification of the HVAC system described in VMP	7 (87.5)	1 (12.5)
Nature and extent of testing and the test procedures following protocols	3 (37.5)	5 (62.5)
Stages of the qualification of the HVAC system followed	4 (50)	4 (50)
Change control procedure followed	7 (87.5)	1 (12.5)
Design condition, operating range, and alert and action limits defined	7 (87.5)	1 (12.5)
Out-of-limit results recorded and their impact investigated	7 (87.5)	1 (12.5)
Maximum time interval between tests defined	6 (75)	2 (25)
Requalification performed when any change occurs	6 (75)	2 (25)
Energy saving procedures	7 (87.5)	1 (12.5)
Documents, i.e., qualification manual and a system of airflow schematic…	7 (87.5)	1 (12.5)
Planned preventive maintenance program	7 (87.5)	1 (12.5)
Maintenance personnel receiving appropriate training	7 (87.5)	1 (12.5)
HEPA filters changed	7 (87.5)	1 (12.5)
Maintenance activity assessed	7 (87.5)	1 (12.5)
Maintenance activities scheduled	7 (87.5)	1 (12.5)

**Table 3:** Implementation of GMP in HVAC systems on the products protection in local pharmaceutical manufacturing companies, 2021.

GMP in HVAC systems on products protection requirements	Yes *N* (%)	No *N* (%)
Areas for manufacture cleaned	5 (62.5)	3 (37.5)
Air filtration and air change rates set	5 (62.5)	3 (37.5)
Operational conditions carried out appropriately	5 (62.5)	3 (37.5)
Materials and products protected from contamination and cross-contamination	3 (37.5)	5 (62.5)
Airborne contaminants controlled through effective ventilation and filtration	4 (50)	4 (50)
External contaminants removed by effective filtration of the supply air	4 (50)	4 (50)
Internal contaminants controlled by dilution contaminants in the room	5 (62.5)	3 (37.5)
Level of protection and air cleanliness for different areas determined	5 (62.5)	3 (37.5)

**Table 4:** Implementation of GMP in HVAC systems on the protection of the environment among local pharmaceutical manufacturing companies, 2021.

GMP in HVAC systems on environmental protection requirements	Yes *N* (%)	No *N* (%)
Exhaust air discharge with adequate filtration	3 (37.5)	5 (62.5)
Less potent powders filtered according to EN779	2 (25)	6 (75)
Harmful substances filtered through HEPA filters according to EN1822	2 (25)	6 (75)
All filter banks provided with pressure differential indication gauges	6 (75)	2 (25)
Filter pressure gauges marked with clean and change out filter resistance	7 (87.5)	1 (12.5)
Monitoring of filters performed at regular intervals	7 (87.5)	1 (12.5)
Reverse pulse dust collectors used	5 (62.5)	3 (37.5)
Exhaust air quality determined	5 (62.5)	3 (37.5)
Fume, dust, and effluent control designed, installed, and operated	6 (75)	2 (25)
Type and quantity of vapors to be treated known	6 (75)	2 (25)

**Table 5:** Implementation of GMP in HVAC systems on the prevention of cross-contamination in local pharmaceutical manufacturing companies, 2021.

GMP in HVAC systems on the prevention of cross-contamination requirements	Yes *N* (%)	No *N* (%)
Pressure cascade regime and direction of airflow appropriate	7 (87.5)	1 (12.5)
Highly potent products manufactured under a pressure cascade regime negative relative to atmospheric pressure	5 (62.5)	3 (37.5)
Pressure cascade for each facility individually assessed	6 (75)	2 (25)
Ceilings and walls, close fitting doors, and sealed light fittings in place to limit ingress or egress of air	6 (75)	2 (25)

**Table 6:** Overall GMP implementation in HVAC systems among local pharmaceutical manufacturing companies, 2021.

Requirements	Implementation status								
	EPMC-01	EPMC-02	EPMC-03	EPMC-04	EPMC-05	EPMC-06	EPMC-07	EPMC-08	Total: *N* (%)
Premises	3	3	2	3	4	3	4	2	24 (75%)
Design and maintenance practices	18	19	16	21	25	22	25	1	147 (70.67)
Products protection	4	5	0	5	8	5	8	—	35 (56.6)
Environmental protection	6	6	4	7	10	6	10	—	49 (61.25)
Prevention of cross-contamination	4	4	2	3	4	3	4	—	24 (75)
Total (%)	35 (67.3)	37 (71.2)	24 (46.2)	39 (75)	51 (98.1)	39 (75)	51 (98.1)	3 (5.8)	279 (67.1)

**Table 7:** Sociodemographic characteristics of respondents in local pharmaceutical manufacturing companies, 2021.

Sociodemographic characteristics of respondents		*N* (%)
Sex	Male	7 (87.5)
	Female	1 (12.5)
	Total	8 (100)

Age of respondents	18–25	0 (0)
	26–35	1 (12.5)
	36–45	5 (62.5)
	46–55	2 (25)
	Total	8 (100)

Current position	Production manager	1 (12.5)
	Technical manager	1 (12.5)
	QA manager	6 (75)
	Total	8 (100)

Experience with the current position	1–5 years	2 (25)
	6–10 years	3 (37.5)
	> 10 years	3 (37.5)
	Total	8 (100)

Total year experience	6–10 years	4 (50)
	> 10 years	4 (50)
	Total	8 (100)

Level of education	MSc	5 (62.5)
	BSc or equivalent	3 (37.5)
	Total	8 (100)

Educational background	Pharmacist	6 (75)
	Chemist	1 (12.5)
	Engineer	1 (12.5)
	Total	8 (100)

## Data Availability

The relevant data are available from the corresponding author on reasonable request.

## Nomenclature

cGMP: Current good manufacturing practice
EFDA: Ethiopian Food and Drug Administration
GMP: Good manufacturing practice
HEPA: High-efficiency particulate air
HVAC: Heat, ventilation, and air conditioning
VMP: Validation master plan
WHO: World Health Organization

## Supporting Information

One supporting file was attached. It is Supporting file 1, which contains the structured observational checklist of GMP implementation on the HVAC system in the Ethiopian pharmaceutical manufacturing companies.
