# Supplementary material for: Compliance of Pharmaceutical Manufacturing Companies to Good Manufacturing Practices in Heating, Ventilation, and Air-Conditioning Systems: The Case of Local Ethiopian Firms
Source: Adv Pharmacol Pharm Sci. 2024 Dec 7;2024:6109415. doi: 10.1155/adpp/6109415 (PMC11646143; doi:10.1155/adpp/6109415)
Supplement: Supporting Information — Additional supporting information can be found online in the Supporting Information section. [file 6109415.f1.docx]

**Structured observational checklist of GMP implementation on the HVAC system in the local Ethiopia pharmaceutical manufacturing companies**

Pharmaceutical industry’s code----------------------------------------

**1: Premises**

| s.no. | **Question** | **Response** | | |
| --- | --- | --- | --- | --- |
|  |  | **Yes** | **No** | **Remark** |
| 1.1. | Does the company have adequate airlocks, i.e., personnel airlocks, material airlocks? |  |  |  |
| 1.2. | Is the design of HVAC system closely coordinated with design of building? |  |  |  |
| 1. 3. | Are detailed diagrams depicting pressure cascades, air flow directions and flow routes for personnel and materials prepared and maintained? |  |  |  |
| 1.4. | Do personnel and materials move from a higher cleanliness zone to a lower cleanliness zone then, back to a higher cleanliness zone? |  |  |  |

**2: Design of HVAC Systems**

| s. no. | **Question** | **Response** | | |
| --- | --- | --- | --- | --- |
|  |  | **Yes** | **No** | **Remarks** |
| 2.1. | Is the HVAC system capacity sufficient to ensure the required performance? |  |  |  |
| 2.2. | Are risk management principles applied during design of HVAC system? |  |  |  |
| 2.3. | Are materials for constructing the components of the HVAC system source(s) of contamination? |  |  |  |
| 2.4. | Are intake and exhaust air terminal positioned one another in manner of preventing cross contamination? |  |  |  |
| 2.5. | Are air conditions and limits for temperature, relative humidity and air cleanliness specified? |  |  |  |
| 2.6. | Are room recovery rate of the HVAC system specified? (i.e., level of room cleanliness, relative humidity, room pressure and microbial limit within specified time) |  |  |  |
| 2.7. | Are air distribution and airflow patterns appropriate and effective? |  |  |  |
| 2.8. | Do you have appropriate alarm system? |  |  |  |
| 2.9. | Are failures of HVAC components assessed? |  |  |  |
| 2.10. | Is the installation system documented as evidence of all measured capacities? |  |  |  |
| 2.11. | Is training provided to personnel after installation and operation of the system? |  |  |  |
| 2.12. | Is the qualification of the HVAC system described in a validation master plan (VMP)? |  |  |  |
| 2.13. | Is the nature and extent of testing and the test procedures follow protocols? |  |  |  |
| 2.14. | Are stages of the qualification of the HVAC system followed? |  |  |  |
| 2.15. | Is a change control procedure followed when changes are planned to the direct impact of HVAC system? |  |  |  |
| 2.16. | Is the design condition, operating range and alert and action limits defined? |  |  |  |
| 2.17. | Are out-of-limit results recorded and their impact investigated? |  |  |  |
| 2.18. | Is the maximum time interval between tests defined? |  |  |  |
| 2.19. | Is requalification done when any change occurs? |  |  |  |
| 2.20. | Is energy saving procedures such as reducing the airflow during non-production hours used? |  |  |  |
| 2.21. | Are documents included in the qualification manuals, system airflow schematics, room pressure cascade drawings, zone concept drawings, air-handling system allocation drawings, particle count mapping drawings? |  |  |  |
| 2.22. | Is there planned preventive maintenance program, procedures and records for the HVAC system? |  |  |  |
| 2.23. | Are the maintenance personnel receiving appropriate training? |  |  |  |
| 2.24. | Are HEPA filters changed either by a trained person, and then followed by installed filter leakage testing? |  |  |  |
| 2.25. | Are any maintenance activity assessed? |  |  |  |
| 2.26. | Are maintenance activities scheduled? |  |  |  |

**3: Protection of Products**

| s.no | **Questions** | **Response** | | |
| --- | --- | --- | --- | --- |
|  |  | **Yes** | **No** | **Remarks** |
| - 1. 5555544 3.1. | Are areas for manufacture exposed to the environment cleaned? |  |  |  |
| - 1. 444444 3.2. | Are air filtration and air change rates set? |  |  |  |
| 3.3. | Are “operational” conditions carried out during the normal production process? |  |  |  |
| 3.4. | Are Materials and products protected from contamination and cross-contamination during all stages of manufacture? |  |  |  |
| 3.5. | Are Airborne contaminants controlled through effective ventilation and filtration? |  |  |  |
| 3.6. | Are external contaminants removed by effective filtration of the supply air? |  |  |  |
| 3.7. | Are Internal contaminants controlled by dilution and flushing of contaminants in the room? |  |  |  |
| 3.8. | Are the level of protection and air cleanliness for different areas determined according to the product being manufactured, the process being used and the product’s susceptibility to degradation? |  |  |  |

**4: Prevention of Cross-Contamination**

| s. no. | **Questions** | **Response** | | |
| --- | --- | --- | --- | --- |
|  |  | **Yes** | **No** | **Remarks** |
| 4.1. | Is the pressure cascade regime and the direction of airflow appropriate to the product and processing? |  |  |  |
| 4.2. | Are highly potent products manufactured under a pressure cascade regime that is negative relative to atmospheric pressure? |  |  |  |
| 4.3. | Is the pressure cascade for each facility individually assessed according to the product handled and level of protection required? |  |  |  |
| 4.4. | Are Ceilings and walls, close fitting doors and sealed light fittings in place, to limit ingress or egress of air? |  |  |  |

5: **Environmental Protection**

| s.no. |  | **Response** | | |
| --- | --- | --- | --- | --- |
|  |  | **Yes** | **No** | **Remarks** |
| 5.1. | Are Exhaust air discharge points on pharmaceutical facilities with adequate filtration? |  |  |  |
| 5.2. | Are less potent powders filtered according to EN779 (Europe new) filtration standards? |  |  |  |
| 5.3. | Are harmful substances the final filters through HEPA filters according to EN1822 filter standards? |  |  |  |
| 5.4. | Are All filter banks provided with pressure differential indication gauges? |  |  |  |
| 5.5. | Are Filter pressure gauges marked with the clean filter resistance and the change out filter resistance? |  |  |  |
| 5.6. | Is Monitoring of filters done at regular intervals? |  |  |  |
| 5.7. | Is reverse pulse dust collectors used? |  |  |  |
| 5.8. | Is the exhaust air quality determined to see the filtration efficiency? |  |  |  |
| 5.9. | Are Fume, dust and effluent control designed, installed and operated? |  |  |  |
| 5.10. | Are the type and quantity of the vapors to be treated known to select the type of filter media as well as the volume of media required? |  |  |  |
